# 

**Published:** 1886-08

**Authors:** 


### BOOKS RECEIVED.

LOCAL ANÆSTHESIA. By J. Leonard Corning, M. D. New York: D. Appleton & Co. 1886.

A COMPEND OF PHARMACY. By F. E. Stewart, M. D., Ph. G. Philadelphia: P. Blakeston, Son & Co. 1886.

OPERATIVE SURGERY OF THE HUMAN BRAIN. By John B. Roberts, M. D. Philadelphia: P. Blakeston, Son & Co. 1886.

MANUAL OF VENEREAL DISEASES. By Berkley Hill, M. D., and Arthur Cooper, M. D. Philadelphia: P. Blakeston, Son & Co. 1886.

THE METHODS OF BACTERIOLOGICAL INVESTIGATION. Translated by H. M. Biggs, M. D. New York: D. Appleton & Co. 1886.

LECTURES ON DIETETICS AND DYSPEPSIA. By Sir William Roberts, M. D., F. R. S. New York: G. P. Putnam Sons. 1886.

BRIGHT'S DISEASE AND ALLIED AFFECTIONS OF THE KIDNEYS. By Charles W. Purdy, M. D. Philadelphia: Lea, Bros. & Co. 1886.

A MANUAL ON AUSCULLATION AND PERCUSSION. By Austin Flint, M. D. New York: D. Appleton & Co. 1886.

DIAGNOSIS AND TREATMENT OF DISEASES OF THE EAR. By Owen D. Pomeroy, M. D. New York: D. Appleton & Co. 1886.

DISEASES OF THE NERVOUS SYSTEM. By James Ross, M. D., LL. D. Philadelphia: Lea Bros. & Co. 1886.

A MANUAL OF PRACTICAL THERAPEUTICS. By E. J. Waring, C. I. E., M. D. Philadelphia: Lea Bros. & Co. 1886.

THE DISEASES OF INFANCY AND CHILDHOOD. By J. Lewis Smith, M. D. Sixth edition. Philadelphia, Pa.: Lea Bros. & Co. 1886.

A MANUAL OF DIETETICS. By J. Milner Fothergill, M. D. New York: William Wood & Co. 1886.

HAND-BOOK OF PRACTICAL MEDICINE. Eichharst. Vol. 1. William Wood & Co., New York. 1886.

WORKS OF HIPPOCRATES. Vol. 1. William Wood & Co., New York. 1886.

DISEASE OF THE SPINAL CORD. Byrom Bromwell. William Wood & Co. New York. 1886.

INSANITY AND ITS TREATMENT. Blandford. William Wood & Co., New York. 1886.

### OUR ADVERTISERS.

NOTICE the change in the advertisement of the Miami Medical College, and write to Dr. J. C. MacKenzie, Secretary, Cincinnati, Ohio, for catalogue.

THE attention of students and others interested is invited to the Medical College advertisement in this issue. Write for catalogue and mention THE JOURNAL.

We desire to direct the attention of our readers to the advertisement of the Font Hill Training School for feeble-minded children on page 28. This school is located upon one of the highest and healthiest points in Howard county, Maryland, thirteen miles from Baltimore. It offers superior advantages. See the advertisement, and write for any information not contained therein to Samuel J. Fort, M. D., P. O. Box 57, Ellicott City, Maryland.

LONG ISLAND COLLEGE HOSPITAL.—Elsewhere will be found the advertisement of this college. It offers superior advantages to students, having a hospital under the immediate control of the Regents and Council of the College. The courses of instruction are given in the hospital, so that the student without loss of time is brought in direct contact with patients, not only in the amphitheater, but also in the wards of the hospital. For full information write to the secretary of the faculty, Dr. J. H. Raymond, Brooklyn, N. Y.

LACTOPEPTINE.—For some years past I have been frequently using lactopeptine manufactured by the New York Pharmacal Association, and it has in every instance given the utmost satisfaction. I have found it to be one of the best artificial digestive preparations, and its value to me has been incalculable in all cases of deficient or troublesome digestion. In dyspepsia, the vomiting of pregnancy, and in cholera infantum it has proved a most excellent remedy.—*Deering J. Roberts, M. D., Prof. Theory and Practice of Medicine, Medical Department University of Tennessee.*

